# Revisiting the connection between Solar eruptions and primary headaches and migraines using Twitter

**DOI:** 10.1038/srep39769

**Published:** 2016-12-23

**Authors:** Staša Milojević

**Affiliations:** 1 Center for Complex Networks and Systems Research, School of Informatics and Computing, Indiana University, Bloomington, IN 47408, United States.

## Abstract

Many internal and environmental triggers of primary headaches have been proposed, but establishing firm evidence for any of them has proved elusive. Geomagnetic storms, the disturbances of Earth’s magnetic field following Solar eruptions, have been proposed as one such trigger. In this study, we utilized a vast amount of self-reported symptoms from the online social networking service Twitter in order to investigate a purported link between the level of geomagnetic activity and the onset of primary headaches and migraines. We analyzed 63 million keyword-bearing messages posted over the three years covering the maximum of Solar Cycle 24. No correlation has been found despite the large sample size. The simulation reveals that the significant correlation would have emerged even if only 1% of headache (2% of migraine) instances were caused by geomagnetic disturbances, thus placing very low upper limits on the prevalence of this trigger among Twitter users.

Headache disorders are among the most prevalent diseases, affecting between 50% and 75% of adults at least once a year^1^,^2^. Tension-type headaches and migraines are the second and third most common diseases globally, afflicting 21% and 15% of the population, respectively. Migraines are among the top ten causes of disability^3^. The relative significance of different proposed endogenous (biological) and exogenous triggers of primary headaches^4^ is not sufficiently understood, and is often confounded^5^,^6^.

The change in meteorological conditions has been a popular candidate for an exogenous trigger, but the link remains inconclusive^7^,^8^,^9^,^10^,^11^. Another proposed exogenous trigger is the geomagnetic activity, and in particular, the geomagnetic storms. Geomagnetic storms have been implicated in a range of health issues^12^,^13^. They represent disturbances in the magnetosphere that result from the stream of particles reaching the Earth 1–2 days after an intense solar eruption^14^. In addition to producing auroras in the Polar Regions, intense storms may disrupt radio communications and satellite operations and cause power outages^15^. Geomagnetic activity is heightened and the storms are more common (up to several per year) around 11-year solar cycle maxima. The evidence for a connection between geomagnetic activity and the frequency or severity of migraines and headaches is inconclusive and is based on small-scale studies (<50 patients)^16^,^17^, none of which covered the periods of maximum solar activity. If such a connection could be more firmly established, it could help us understand the mechanism underlying headache disorders and provide patients with an opportunity to prepare for the onset of symptoms.

We studied the connection between geomagnetic activity and headache/migraine prevalence using Twitter messages collected over a three-year period covering the maximum of the current solar cycle^18^. With over 350 million active users per month, Twitter presents an attractive health-study platform, providing reliable self-reported data at an unprecedented scale^19^ and in real time^20^,^21^,^22^,^23^. Twitter has been used to track outbreaks of infectious diseases^24^,^25^,^26^ and to monitor mood rhythms of millions of people around the world^27^. Twitter was also used to evaluate headache and migraine experiences, most notably their periodicities and patterns of communications about them^21^,^22^.

## Results

A total of 5.6 × 10^7^ and 7.1 × 10^6^ Twitter messages (“tweets”) referring to headaches and migraines, respectively, were collected from November 2012 to January 2016. Forwarded messages and bulk postings were removed. The raw reporting rates ranged from 0 to 150 messages per minute for headaches, and from 0 to 20 messages per minute for migraines. The average daily volume was 51,100 messages pertaining to headaches and 6500 to migraines. We confirmed that the majority of tweets reported actual, real-time symptoms (75% for headaches, 65% for migraines) in accordance with previous estimates^22^.

**Figure 1:**
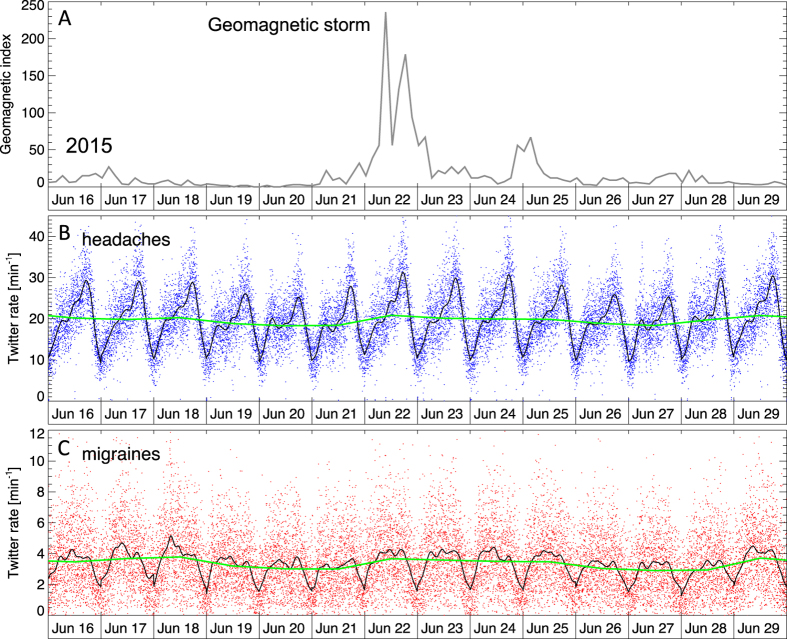
Rate of Twitter messages pertaining to headaches (**B**) and migraines (**C**) during the two-week period surrounding the most intense geomagnetic storm of the current solar cycle, peaking on June 22, 2015. Panel A shows the values of the global geomagnetic index *ap*. Panels B and C show Twitter rates (messages per minute) for headaches (blue dots) and migraines (red dots), respectively. Black curves are smoothed trends and green lines the daily averages. No enhancement in symptoms reporting rate is seen in relation to the storm.

The most intense geomagnetic storm of the current solar cycle (Cycle 24) occurred on June 22, 2015, with the maximum value of global (planetary) 3-hr index (*ap* index) of 230. The index measures the disturbance of the Earth’s magnetic field near its surface. For a two-week period surrounding this geomagnetic storm, Fig. 1 shows geomagnetic activity in panel A, Twitter rates for headaches in panel B, and rates for migraines in panel C. Smoothed trends are overlaid in black, and show a diurnal variation that is consistent with the majority of users being located in North America. Daily averaged rates (green curve) show relatively small fluctuations on these timescales. There are no conspicuous enhancements of Twitter rates corresponding to the storm, on diurnal or intradiurnal timescales.

**Figure 2:**
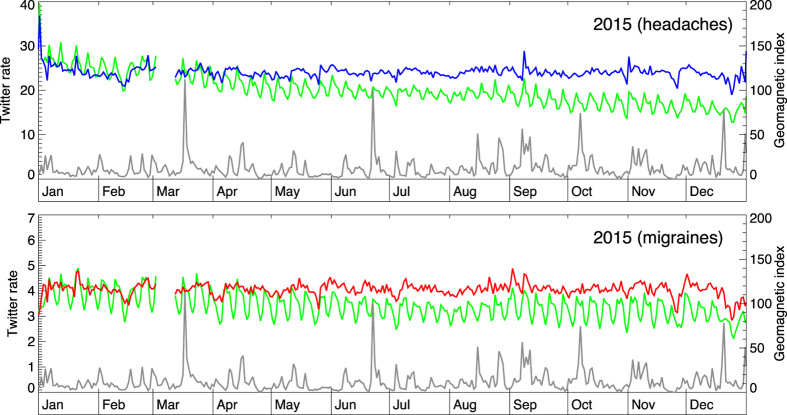
The change in geomagnetic activity (daily averages; gray curve) and the daily rate of Twitter messages (green lines) pertaining to headaches (up) and migraines (down) for year 2015. Blue and red lines show normalized daily rates that account for the weekly modulation and the long-term trend of declining Twitter usage. There is no discernible link between geomagnetic events and symptom reporting.

**Figure 3:**
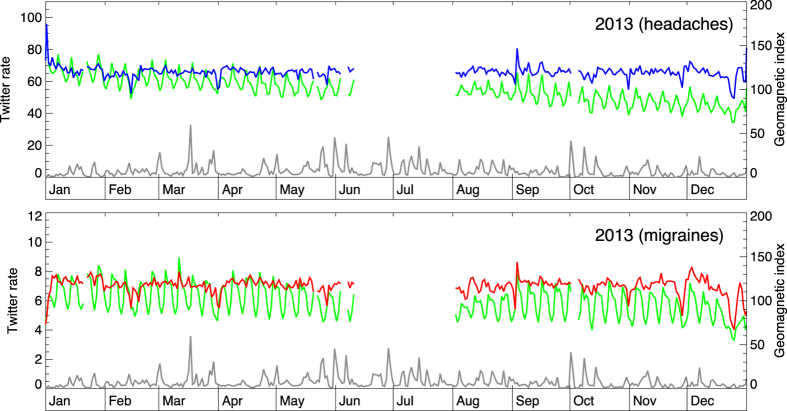
The change in geomagnetic activity (daily averages; gray curve) and the daily rate of Twitter messages (green lines) pertaining to headaches (up) and migraines (down) for year 2013. Period of missing data pertains to Twitter API server change that required the modification of the harvesting software that was not immediately available.

**Figure 4:**
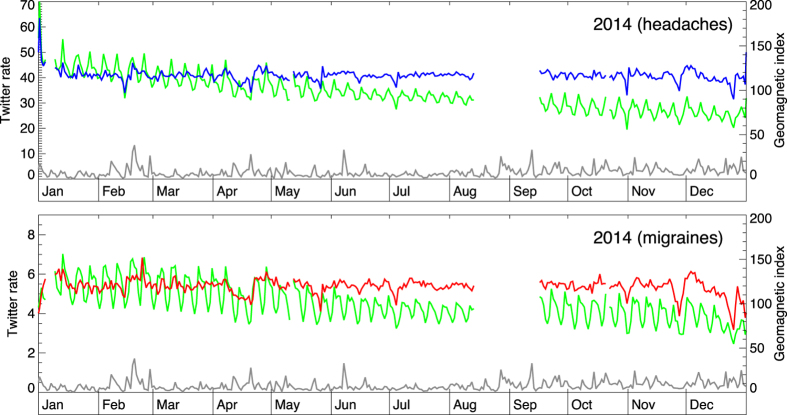
The change in geomagnetic activity and the daily rate of Twitter messages pertaining to headaches (up) and migraines (down) for year 2014. The gap in coverage pertains to a period when Twitter data were not being delivered from the served in full.

In Fig. 2 the scope is extended to a full year (2015). The daily reporting rates for headaches (panel A) and migraines (panel B) (green curves) are shown alongside the daily-averaged geomagnetic index (gray line). Twitter rates exhibit weekly periodicity, as well as a long-term trend of decreasing usage. Normalized Twitter rates, shown as blue and red curves, for headaches and migraines respectively, account for these trends to reveal residual variation. The normalized rates have ~10% day-to-day variation, but no apparent correlation with the peaks of geomagnetic activity. Similar conclusions are reached for years 2013 and 2014 (Figs 3 and 4).

**Figure 5:**
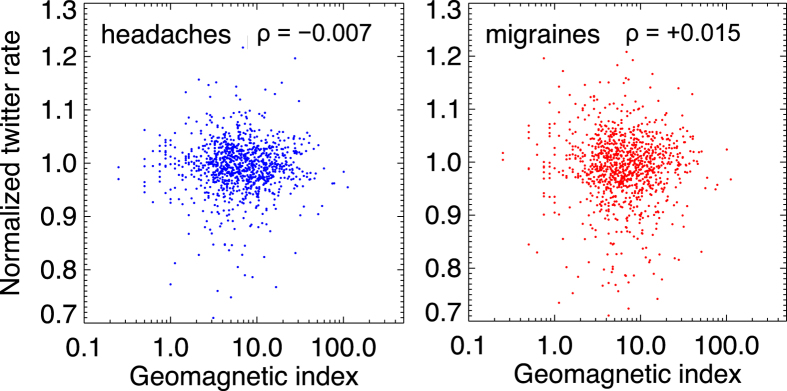
Direct comparison of symptom-reporting rates and the strength of geomagnetic events, for headaches (left) and migraines (right). Spearman correlation indices are low (indicated in panels), consistent with no correlation.

A formal analysis was further performed to check for a potential presence of a weaker correlation, one that would not be discernible by the visual inspection of trends in Figs 2, 3 and 4. In Fig. 5 the normalized daily rates for the entire period of the study (3 years) are plotted against the logarithm of daily-averaged geomagnetic indices. The Spearman correlation indices are −0.007 for headaches and 0.015 for migraines, consistent with no correlation (*P* = 0.82 and 0.74, respectively). No significant correlation is obtained also if the symptoms are allowed to lag behind the geomagnetic events for up to a week.

**Figure 6:**
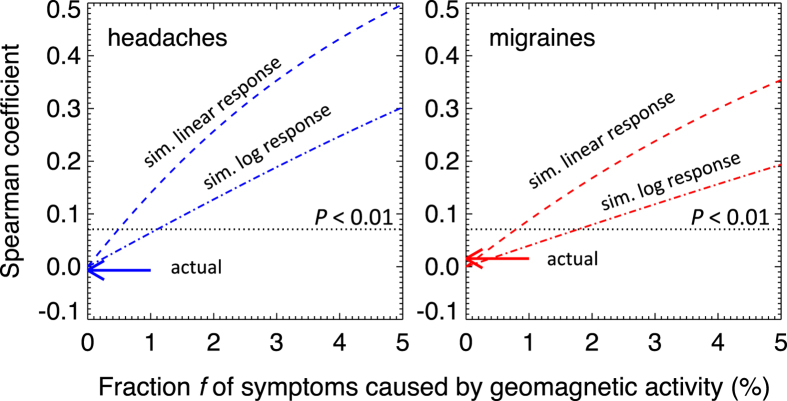
The sensitivity of the data and the method. The graphs show the strength of the correlations after injecting an artificial signal in the data whereby some fraction of headache (left) and migraine (right) instances is due to geomagnetic activity. We model the response as either linear (a stronger response) or logarithmic (a weaker response). Regions above the dotted lines correspond to correlations that are statistically significant (*P* < 0.01). The intersect between the dotted and dashed lines shows that the data/method would have produced significant correlations even if only 1–2% of symptom instances were geomagnetic in origin. The actual correlation coefficients are consistent with 0% (arrows).

In order to determine the confidence interval of our null result, i.e., the sensitivity of the data and the method, we perform the following simulation. We inject an artificial signal in the data whereby some fraction *f* of the symptom instances follows geomagnetic activity, either linearly (a stronger response) or logarithmically (a weaker response). The simulation also takes into account the fraction of tweets that do not correspond to actual symptoms. Figure 6 shows the values of the Spearman correlation coefficients for different values of *f*, based on these two bracketing models of response. It shows that for a correlation to be confirmed at 99% confidence level (*P* < 0.01; above the dotted line), the prevalence of headaches would have to be f > 0.5% to 1.1%, and the prevalence of migraines *f* > 0.8–1.8%. In other words, our results are consistent with zero rate, with upper 99% confidence intervals (*f*) between 1 and 2% (the lower limits are zero). These sensitive upper limits are the result of large sample sizes and extensive temporal coverage.

## Discussion

We analyzed 63 million messages on headaches and migraines posted over the three years period and daily levels of disturbance of the Earth’s magnetic field near its surface to investigate a link between the occurrence of primary headaches and geomagnetic activity. Our main finding is that there is no correlation between the occurrence of primary headaches as self-reported on social network and geomagnetic activity, including geomagnetic storms. Thus, the article contributes evidence against a causation of both headaches and migraines by geomagnetic activity.

This paper contributes to the growing body of literature utilizing traces of online behavior, especially on social media such as Twitter, to address medical and health-related questions. Previous studies on migraines using Twitter have already established that “online behavior is a rich, free source of voluntary self-reports that can enrich or supplement epidemiological and diary studies on disease”^21^. Our findings regarding periodicities of headaches and migraines are in agreement with the findings in other studies^21^. Our study goes further by analyzing a role of a single trigger, the solar activity, in depth.

This study raises several well-known methodological issues related to the usage of Twitter as a health study platform. The first one is related to a population. According to the Pew Internet & American Life Project, between 16% and 23% of adult Americans used Twitter in the period we covered^28^. This fraction varies significantly by age group. It is 31% for 18–29 age group as opposed to 5% for 65+. While our findings show that data/method would have produced significant correlation even if only 1–2% of symptoms reported by Twitter users were geomagnetic in origin, we cannot rule out that certain segments of the population, such as children or seniors, who are not well represented in the data, may be more susceptible to solar activity as a trigger. Furthermore, in our study we have no information on the severity of the symptoms, and the only threshold is that the users feel they should tweet about it. Thus, the population included in our study may be wider, including patients with less severe symptoms than would have been included in traditional, targeted studies. Finally, our findings pertain to the average localities of Twitter users, not taking into account possible differences with geographic location, and especially the latitude. It would be worth exploring whether the population closer to the Polar circle is more susceptible to this trigger. It should be noted, however, that only 2% of Tweets carry information on geographic location, greatly reducing the available sample.

We have shown on a much larger scale than any of the previous studies carried on this topic so far (63 million instances vs. fewer than 50 individuals) that there is no correlation between geomagnetic storms and headaches and migraines, demonstrating the power of social networking data as a health study platform. Further in-depth studies using a wide variety of data sources would be recommended to deepen our understanding when it comes to solar activity as a trigger for headaches and migraines. For example, it would be interesting not only to focus on particular segments of population, but to examine suites of triggers simultaneously, rather than focusing on a single trigger at a time. Such studies can not only improve the knowledge of onset of symptoms, helping patients in management, but also shed light on the complex nature of mechanisms leading to one of the most widely spread disorders of contemporary world.

## Materials and Methods

Global (or planetary) geomagnetic index *ap* is reported in 3-hr intervals (8 measurements per day) as a weighted average from 13 stations across the world. This index is a linear version of *K*_*p*_ index, introduced in 1939^29^. We have obtained *ap* index values from World Data Center for Geomagnetism in Kyoto, based on the central repository at Geo Forschungs Zentrum (GFZ) in Potsdam.

We have used *Webometric Analyst* to harvest the tweets containing the following keywords and phrases: ‘headache[s]’, ‘head ache[s]’, ‘migraine[s]’, ‘pain in my head’. The searches would deliver essentially all the messages containing these keywords (i.e., the searches were not subject to volume restrictions), except during intermittent dropouts in internet connectivity or server availability. Data collection commenced on November 22, 2012 and was terminated on February 5, 2016. The data covered the maximum of Solar Cycle 24.

Message IDs were checked to eliminate duplicate entries. Furthermore, all re-tweeted messages were removed. Occasionally (once a month), a large number of bulk (“spam”) messages would appear over the durations of minutes to an hour. These messages tended to be long (40–140 characters) and contained essentially identical content, usually promoting some online content or service. We removed all messages which are longer than 40 characters if their content (except for the URL addresses or usernames, which usually differ from one spam posting to another) is identical. Note that the spline-fitting process (discussed below) would not be affected by short-lasting spikes in counts regardless. The above processing left 62.9 × 10^6^ tweets (55.8 × 10^6^ on headaches and 7.1 × 10^6^ on migraines).

We randomly selected ~200 messages on headaches and ~200 on migraines, and inspected their contents to determine the fraction of messages (*r*), that actually pertain to real-time symptoms, as opposed to a figurative usage of a word headache, or a reference being made to headaches and migraines in general, e.g., a news item about them. Inspection was performed by two independent reviewers, who obtained the same fractions: *r = *75% for headaches and *r = *65% for migraines. Cohen’s κ coefficient which measures inter-coder reliability taking into account chance agreement is κ = 0.81 for headaches and κ = 0.75 for migraines, which can be characterized as excellent^30^. Our estimate for migraines also agreed remarkably well with the content analysis of over 20,000 migraine-related tweets, that found that 64.5% of them are reporting on real-time migraine attacks^22^.

We defined the reporting rate as the number of tweets in 1-minute increments. Rates pertaining to a single day (1440 minutes) were iteratively fitted with a cubic spline in order to interpolate over any minutes in which the tweets were missing due to internet/server interruptions (~2% of minutes), and to produce a smooth trend. The average rates pertaining to headaches were sufficiently high (35 min^−1^) that their minute-to-minute fluctuation followed a normal distribution, and outliers could thus be identified as minutes whose rates deviated more than 3 standard deviations from the fit. Outliers were excluded, leaving minutes with complete data, and the function was re-fitted. Five iterations were carried out, resulting in a converged fit. For migraines, where the low reporting rates prevented the application of the above procedure, the fit was performed using the minutes that were identified as having complete data for headaches. The cubic spline fit preserved all the salient features of the intradiurnal change in the reporting rate (Fig. 1). The fitting was performed for each day separately. Counts overlaid with fits were inspected to assess their robustness. Days where large chunks of data were missing and could not be accurately interpolated over were identified and removed from the subsequent analysis. The beginning of the day (for both the Twitter analysis and geomagnetic activity) was taken at 1000 UTC (0500 EST), as it corresponds to the minimum in Twitter activity.

For days with robust data and fits (1060 days = 2.9 years), the average of the fit was calculated to get the daily reporting rate. The trend of daily rates is presented in Figs 2, 3 and 4. It shows a gradual long-term decline in the reporting rates, as well as a variation with a 7-day periodicity. To normalize the long-term trend, we fitted a smooth function (also a cubic spline) over all 3-years and divided the daily counts by the fit. The weekly periodicity, whether it reflects the variation in the likelihood of posting to Twitter or the actual change in the prevalence of symptoms, may mask subtle correlations with geomagnetic activity. The rates were de-trended for weekly variation in the following way: Each day was normalized to the typical reporting rate for that day of the week, which was determined by taking the medians of rates of the same day of the week four weeks before and four weeks subsequent to the day to be normalized. For example, the counts for Monday, November 2 are normalized with respect to the median of Monday rates from October 5 to November 30, excluding November 2. We tested different sampling windows and found that 4 weeks produced the smallest standard deviation in de-trended values.

De-trended, normalized rates were analyzed for the presence of a correlation with respect to the daily-averaged geomagnetic index using Spearman correlation test, but no significant correlation was found (Fig. 3). In order to determine how strong a signal must be before a correlation becomes statistically significant, we performed a simulation in which we injected an artificial signal into the normalized rates, such that fraction *f* of tweets pertaining to actual symptoms were directly modulated by geomagnetic activity, either by following a stronger, linear, or a weaker, logarithmic response. Simulated rate *N*_*i*_, for day *i* is:


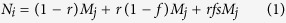


where *M*_*j*_ is the actual rate of randomly picked day *j*, *r* is the known fraction of tweets reporting actual symptoms and *s* is the response function:


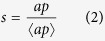


for linear response, or


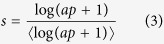


for logarithmic response. The first term in *N*_*i*_ are non-symptom tweets, the second term are symptom tweets that originate from causes other than geomagnetic activity, and the third term are symptom tweets due to geomagnetic activity. If there were no changes in geomagnetic activity (*s* = 1), or if *f* was 0, the simulated rates become just the actual (but shuffled) rates.

## Additional Information

**How to cite this article**: Milojević, S. Revisiting the connection between Solar eruptions and primary headaches and migraines using Twitter. *Sci. Rep.* **6**, 39769; doi: 10.1038/srep39769 (2016).

**Publisher's note:** Springer Nature remains neutral with regard to jurisdictional claims in published maps and institutional affiliations.
